# Exploring the Spatial-Temporal Microbiota of Compound Stomachs in a Pre-weaned Goat Model

**DOI:** 10.3389/fmicb.2018.01846

**Published:** 2018-08-15

**Authors:** Yu Lei, Ke Zhang, Mengmeng Guo, Guanwei Li, Chao Li, Bibo Li, Yuxin Yang, Yulin Chen, Xiaolong Wang

**Affiliations:** College of Animal Science and Technology, Northwest A&F University, Xianyang, China

**Keywords:** taxonomic diversity, ruminant, microbial community, rumen microbiology, gut microbiota

## Abstract

Ruminant animals possess a characteristic four-compartment stomach (rumen, reticulum, omasum, and abomasum) that is specialized for pre-intestinal digestion of plant materials. Of these four compartments, the rumen is the largest. The rumen’s diverse microbial community has been well studied. However, the current understanding of microbial profiles in the reticulum, omasum and abomasum are lacking. In the present study, fluid samples from the reticulum, omasum, and abomasum of goats at 3, 7, 14, 21, 28, 42, and 56 days after birth, as well as the negative controls (NC) used for microbial DNA extraction, were subjected to 16S rRNA sequencing. By filtering operational taxonomic units (OTUs) in NC, distinct temporal distributions of microbes were observed in the different compartments, we showed that the OTUs in control samples had a large effect to the samples with low microbial density. In addition, Proteobacteria gradually decreased with age from days 3 to 56 in all three compartments, and the relative abundance of Bacteroidetes increased from 24.15% (Day 3) to 52.03% (Day 56) in abomasum. Network analysis revealed that *Prevotellaceae_UGG-03* and *Rikenellaceae_RC9* were positively correlated with *Prevotella_1*, lending support to the well understood fact that cellulose is well digested in compound stomachs prior to the rumen. Pathway analysis revealed that gene expression in abomasum at Day 3 were primarily related to Glycolysis/Gluconeogenesis and Pyruvate metabolism, suggesting that colostrum digestion is the dominant function of the abomasum at an early age. These findings combined with other recent rumen microbiota data show that the microbiome landscape represents three distinct stages in ruminant stomachs. The first stage is to gain access to external microorganisms at Day 0–14, the secondary stage is for microbial transition at Day 14–28, and the third stage is for exogenous and endogenous microbial colonization beyond Day 28 of age. Our results provide insight into microbiota dynamics in ruminant stomachs, and will facilitate efforts for the maintenance of gastrointestinal balance and intervention with starter diets in juvenile ruminants during early development.

## Introduction

Microbiota of mammalian gastrointestinal tracts (GIT) are complex ecosystems which are composed of diverse microbial populations, including bacteria, archaea, ciliate protozoa, and anaerobic fungi (Falony et al., 2016). Maternal animals are subjected to three major physiological stresses, including gestation, parturition, and nursing. Of them, stresses of nursing occur during the pre-weaning stages (e.g., 60 days after birth in small ruminants such as goats and sheep) and is the most important physiological stage for kids. After birth, the main colostrum or mature milk substitute is liquid feed administered along the esophageal ditch directly into the abomasum and intestines of ruminants, which is similar to other monogastric animals (Khan et al., 2011). Studies have demonstrated that colostrum and mature milk are an important source of gut bacterial colonization, accounting for the introduction of >200 species (Toscano et al., 2017). This has a strong impact on the newborn’s health for several months after birth (Fernández et al., 2013; Toscano et al., 2017). As solid feed intake increases, the rumen transitions to its mature role in making important physiological nutrients bioavailable. At this stage, the kids have transitioned from non-ruminant to ruminant, and the nutrient digestion and metabolism pathways undergo a qualitative change (Rey et al., 2012). In addition, the rumen and the other stomach compartments (reticulum, omasum, and abomasum) serve as important sites of colonization for many commensal microorganisms (Peng et al., 2015). Although studies of ruminal microorganisms have resulted in an improved understanding of the composition and colonization of rumen microbial communities (Jami et al., 2013; Mao et al., 2015; Morgavi et al., 2015; Wang et al., 2016), the dynamics of microbiota development in the three other stomach compartments has been severely understudied.

Digestion in ruminants is characterized based on each of the four stomach compartments. Three of them are known as the forestomaches, which consist of the rumen, reticulum, and omasum (Jonsson, 2011). The forestomaches contain large numbers of microorganisms that aid in the anaerobic degradation of nutrients. The forestomach microorganisms are capable of breaking bonds in cellulose and hemicellulose, which are major energy sources for ruminants. Once the partially digested feed has passed through the forestomaches, it enters the abomasum, which is similar to the stomach in monogastric animals. Up until approximately 3 weeks of age, the rumen is largely non-functional. Development of the rumen undergoes a process of development spanning roughly the first 6 months of life, at which point it becomes fully functional (Trenkle, 2001). In contrast, the lower gastrointestinal tract can develop a functional microbiome within a few weeks (Morowitz et al., 2010). In addition, fermentation in forestomaches produces a variety of end products which are subsequently absorbed in the digestive tract. Fermentation of a fiber-rich diet is a time-consuming process (Animut and Goetsch, 2008), whereas in hay-fed goats, the rumen plays a major role in the digestion of a fiber-rich diet, but in milk-fed kids, digestion occurs mainly in the abomasum.

The temporal sequence of microbial establishment in ruminant stomachs at early developmental stages holds great promise for the economical rearing of replacements, as well as for the host’s well-being. We have previously investigated microbial communities in the rumen of weaned (Han et al., 2015) and pre-weaned goats (Zhang et al., 2018, unpublished). We observed that the colonization of microorganisms is highly correlated to the function in the rumen. The composition of the primary bacterial communities is determined and acquired shortly after birth not only in the rumen, but also in the other stomach compartments of ruminants. The spatial-temporal patterns occurring in these communities remain largely unknown. In this study, 16S rRNA sequencing was used to investigate the colonization of reticulum, omasum, and abomasum microbes in pre-weaned goats as a ruminant model. We sought to determine which developmental stages have significant effects on microbiota communities in the different stomach compartments. This study provides new insights into bacteria communities in pre-weaned goats, which may be useful in designing strategies to promote colonization of target communities.

## Materials and Methods

### Animal Handling and Sample Collection

All sampling of animals was approved by the Institutional Animal Care and Use Committee of the Northwest A&F University under permit number 2014ZX08008002. All surgeries were performed while animals were anesthetized with xylazine chlorhydrate. All efforts were made to minimize animal suffering.

Two months prior to sample collection, pregnant does were raised at the experimental facilities of the Shaanbei Cashmere Goat Farm (Hengshan, Shaanxi). After delivery, the single kids were housed together with their mothers for nursing. The mother’s milk was the sole food until Day 25. Between Day 25 and Day 56, kids were provided granule feed and high-quality alfalfa twice daily at 09:00 and 18:00 (not limit the feed intake of the kid during feeding process). The ingredients and nutrient composition of the diets are summarized in **Supplementary Table S1**. Fresh water was provided for *ad libitum* consumption throughout the experimental period. Three kids were slaughtered at each experimental time point (Day 3, Day 7, Day 14, Day 21, Day 28, Day 42, Day 56). The reticulum, omasum, and abomasum contents were collected into 20 mL cryopreservation tubes. The sampled liquid was and immediately stored at −80°C for further analysis as previously reported (Zhang et al., 2018, unpublished).

### DNA Extraction, PCR Amplification, and Sequencing

Microbial DNA was extracted from reticulum, omasum, and abomasum fluid samples using the Fast-DNA^®^ Spin kit for soil (Bio101, Vista, CA, United States) according to manufacturer’s instructions. During the study, we strictly controlled the test samples and negative controls (NC) were set during the DNA extraction and amplification process. To ensure the fluid samples were not contaminated by reagents used for DNA extraction, two commercial kits to isolate DNA from fluid and NCs (ddH_2_O and reagents used for DNA extraction) were used (**Supplementary Figure S1**), these extracted DNA were further amplified (**Supplementary Figure S1**). These results collectively indicated we found that the stomach fluid DNA samples were not contaminated during the DNA extraction process, thereby confirming the reliability of samples used in this study (**Supplementary Figure S1**). We were also unable to extract DNA from the Day 0 samples in reticulum due to inadequate fluids. Additionally, the NC, DNA-free water and buffer used for DNA extraction, were subjected to paired-end sequencing (2 × 250/bp) using the Illumina HiSeq2500 platform. The R package “decontam”^1^ was used to identify contaminants in the metagenomic sequencing data (Davis et al., 2017). In addition, the V3-V4 hypervariable region of the bacteria were amplified by PCR using the following thermocycling protocol: 95°C for 2 min, followed by 30 cycles at 95°C for 20 s, 55°C for 30 s, and 72°C for 30 s, followed by a final extension at 72°C for 5 min. The primers used to amplify the sequences were as follows: V341F (5′-CCTAYGGGRBGCASCAG-3′) and V806R, (5′-GGACTACHVGGGTWTCTAAT-3′). A molecular barcode in the form of an 8-base sequence unique to each sample was added for subsequent identification and processing. PCR reactions were performed in triplicate 20 μL reactions containing 5 μL of 5× FastPfu Buffer, 2 μL of 2.5 mM dNTPs, 0.8 μL of each primer (5 μM), 0.4 μL of FastPfu Polymerase, and 10 ng of template DNA. The resulting amplicons were resolved on 2% agarose gels. The resulting bands were extracted and purified from the gels using the AxyPrep DNA Gel Extraction Kit (Axygen Biosciences, Union City, CA, United States) according to the manufacturer’s instructions. The resulting products were quantified using the QuantiFluor^TM^ -ST kit (Promega, United States). Sample libraries were pooled in equimolar ratios, and were subjected to paired-end sequencing (2 × 250/bp) using the Illumina HiSeq platform according to a standard protocol at the Mega Genomics Company Limited, Beijing, China. Amplicon sequences generated in this study are available at the NCBI-SRA under accession: SRP090491.

### Data Analysis

Raw fastq files were de-multiplexed, and quality-filtered using QIIME (version 1.9.1) (Caporaso et al., 2010) with the following criteria: 300 bp reads were truncated at any site with an average quality score <20 over a 50 bp sliding window; truncated reads shorter than 50 bp were discarded; exact barcode matching; two nucleotide mismatch in primer matching; reads containing ambiguous characters were removed; sequences with at least a 10 bp overlap were assembled according to their overlap sequence. Reads which could not be assembled were discarded. Based on the overlapping sequences, paired-reads were merged in to a single read. The merged reads were then used for operational taxonomic unit (OTU) clustering, taxonomic classification, and community diversity assessment. The resulting microbial communities were used for comparison of similarity or dissimilarity between different sample groups, analysis of the relationships between microbial communities and environmental factors, phylogenetic analysis, as well as other statistical analyses. In this study, total 4,481,828 raw tags were obtained, among them 4,106,574 effective tags were used for further analysis.

For phylogenetic analysis, OTUs were clustered with a cutoff of at least 97% similarity using UPARSE (version 7.1^2^) (Blaalid et al., 2013; Edgar, 2013). Chimeric sequences were identified and removed using UCHIME. In ecology, alpha diversity (α-diversity) is the mean species diversity in sites or habitats on a local scale. These OTUs were used for the determination of α-diversity (Shannon and Simpson) and richness (Ace and Chao) (Schloss et al., 2011; Quast et al., 2012; Wang et al., 2012; Amato et al., 2013). Principal component analysis (PCA), PLS-DA (Partial Least Squares Discriminant Analysis) and species correlation network were analyzed on the free online platform of Major bio I-Sanger Cloud Platform ^3^.

The taxonomy of each 16S rRNA gene sequence was analyzed using RDP Classifier^4^ against the Silva (Cole et al., 2008) (SSU123) 16S rRNA database using a confidence threshold of 0.7. Functional gene annotations were predicted using FGR (Release7.3^5^) (Cole et al., 2013). Based on the relative abundance of the microorganisms, those in high abundance or those that were artificially selected in the sample comparison between two or more groups were subjected to a Welch’s test to determine statistical significance using the R “gplots package.” Significant differences between taxa were assessed by Tukey’ test comparison procedure using *SPSS* version 22.0 for Windows.

### Prediction of COG Function Classification and Functional KEGG Pathways

PICRUSt (Langille et al., 2013) was used to normalize the OTU table by the 16S rRNA copy number predictions. Consequently, the OTU abundances more accurately reflects that of the organisms in the population. Metagenomic predictions were then made by looking up the re-calculated genome content for each OTU, and then multiplying the normalized OTU abundance by each KEGG ontology (KO) abundance in the genome, and summing the KO abundances for each sample. The resulting predictions yielded a table of KO abundances for each metronome sample in the OTU table (White et al., 2009; Segata et al., 2011).

## Results

### Diversity, Richness, and Composition of the Bacterial Communities in Compound Stomachs

A total of 67 samples, representing the reticulum, omasum, and abomasum from 21 goats (*n* = 3) and NC including DNA-free water and buffer (*n* = 4) were used for DNA extraction. Sequencing of the16S rRNA samples from these stomach liquids generated 4,181,452 clean tags with an average of 66,372 clean tags per sample (**Supplementary Table S2**), and base quality values with a Q30 ratio greater than 83.16% (**Supplementary Table S2**). Four NC samples were sequenced to generate 105 OTUs (**Supplementary Table S3**), the 16S rRNA samples from NC generated 16,939 clean tags with an average of 4,234 clean tags per sample. The NC sequences were eliminated for subsequent analysis with the R package “decontam” (see text footnote 1). The result further demonstrates that it is critical to pay assiduous attention to controls, when sequencing samples were in a low microbial density. A rarefaction analysis including all samples revealed a curve approaching saturation (**Supplementary Figure S2**), and the Chao1 index indicated that approximately 98% of microbial genes were captured in the samples. The Chao1 index of the OTU level indicated that there was a significant difference in the α diversity index between the various age groups, except for Day 3 and Day 7 (**Table 1**). These data suggest that the communities exhibited a higher α diversity with increasing age.

**Table 1:** Alpha diversities within each age group in bacteria, richness, and evenness were measured by the Chao1 evenness index.

Time	Reticulum	Omasum	Abomasum
Day 3	433.78^a^	362.28^a^	321.26^a^
Day 7	525.27^ab^	528.28^ab^	392.21^a^
Day 14	805.07^bc^	659.58^bc^	503.03^a^
Day 21	859.2^d^	871.67^de^	869.06^b^
Day 28	803.96^d^	982.83^e^	785.42^b^
Day 42	637.20^cd^	737.06^cd^	724.02^b^
Day 56	724.38^d^	822.95^cde^	845.39^b^

*Tukey’ test for Chao index of OTU level in reticulum, omasum, and abomasum. a, b, c, d, e Means with different superscripts differ significantly each column.*

### Analysis of Bacterial Composition in Reticulum

**FIGURE 1:**
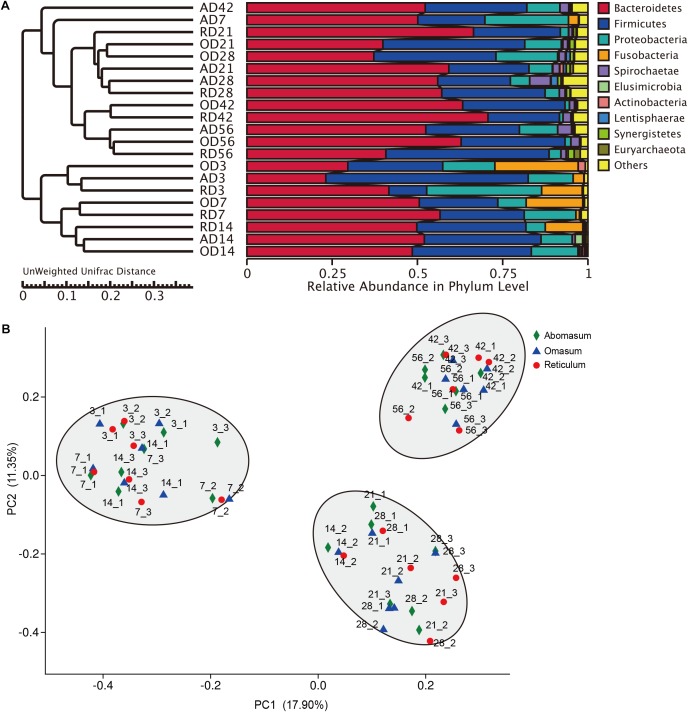
Distribution of bacterial composition in goat compound stomachs. **(A)** UPGMA (Unweighted Pair-group Method with Arithmetic Mean) analysis of the similarities between different samples, and cluster analysis was used to construct the clustering tree of samples. the UPGMA cluster tree based on the Unweighted Unifrac distance. **(B)** Principal coordinate analysis (PCoA) profile of microbial diversity across all samples using an unweighted UniFrac metric, the percentage of variation explained by PC1 and PC2 are indicated in the axis.

From days 3 to 56, the proportions of Proteobacteria gradually decreased with age (*P* = 0.039) at the phylum level. The majority of annotated reads (45.46%) belonged to Bacteroidetes, followed by Proteobacteria (27.29%) at Day 3. At Day 7, Bacteroidetes (57.97%) comprised the dominant phyla, followed by Firmicutes (24.47%). However, at D56, Firmicutes (46.74%) and Bacteroidetes (42.29%) accounted for the majority of annotated reads (**Figure 1A** and **Supplementary Table S4**). At the genus level, *Mannheimia*, *Bacteroides*, *Fusobacterium*, and *Porphyromonas* were detected as the dominant genera at Day 3, but the ratios of these genera gradually decreased with increasing age (**Table 2**). On the contrary, *Prevotella_1*, *Rikenellaceae_RC9*, *Ruminococcus_2*, *Bacteroidales_S24-7* were relatively low in samples collected from the Day 3 group but became the most abundant genera in samples collected after Day 28 (**Table 2** and **Supplementary Table S5**). In addition, the annotated reads (about 1%) belonged to *Ruminococcaceae_UCG-005* at days 21 and 28, but were essentially absent at other ages (**Table 2**). Therefore, the microbiota community in reticulum data demonstrates a strong temporal specificity.

**Table 2:** Phylum and genus level composition in goat compound stomachs.

Tissue	Species	D3 (%)	D7 (%)	D14 (%)	D21 (%)	D28 (%)	D42 (%)	D56 (%)	*P*-value
Reticulum	p_Proteobacteria	27.290^a^	15.250^ab^	5.523^b^	2.915^b^	4.162^b^	1.034^b^	3.688^b^	0.039
	*g_Porphyromonas*	14.840^a^	8.238^a^	13.210^a^	6.339^a^	0.226^b^	0.016^b^	0.010^b^	0.003
	*g_Rikenellaceae_RC9*	0.005^a^	0.364^a^	0.446^a^	14.780^b^	7.339^ab^	3.240^a^	6.662^ab^	0.003
	*g_Ruminococcus_1*	0.005^a^	0.844^a^	0.523^a^	0.373^a^	1.255^b^	1.643^b^	2.570^b^	0.003
	*g_Desulfovibrio*	0.015^a^	0.017^a^	0.087^a^	0.464^a^	0.218a	0.252^a^	2.089^b^	<0.001
	*g_Ruminococcaceae_UCG-005*	0.002^a^	0.167^ab^	0.028^a^	1.444^b^	1.175^bc^	0.108^ac^	0.464^abc^	<0.001
Omasum	*g_Prevotella_1*	0.012^a^	0.062^a^	1.125^a^	1.794^a^	5.493^a^	27.430^b^	19.580^c^	0.001
	*g_Christensenellaceae_R-7*	0.077^a^	1.083^a^	4.730^ab^	9.158^b^	5.712^ab^	1.323^a^	0.980^a^	0.009
	*g_Ruminococcaceae_NK4A214*	0.051^a^	2.487^ad^	8.075^b^	7.405^c^	5.804^d^	0.994^a^	0.937^a^	0.012
	*g_Prevotellaceae_UCG-001*	0.003	2.529	2.095	2.443	0.457	4.440	8.60	0.013
Abomasum	*g_Ruminococcaceae_NK4A214*	0.162^a^	2.437^a^	10.590^b^	4.933^b^	4.278^b^	1.667^a^	1.890^a^	<0.001
	*g_Rikenellaceae_RC9_gut*	0.003^a^	0.218^a^	0.249^a^	6.488^b^	5.511^b^	2.110^a^	3.010^a^	0.001
	*g_Prevotella_1*	0.008^a^	0.044^a^	0.625^a^	7.856^a^	9.909^ac^	25.940^b^	24.580^c^	<0.001
	*g_SP3-e08*	0.001^ac^	0.003^ac^	0.004^ac^	7.566^b^	2.576^c^	0.152^ac^	0.292^ac^	0.005
	*g_Conchiformibius*	3.570^a^	0.340^b^	0.210^b^	0.060^b^	0.001^b^	0.001^b^	0^b^	<0.001

*The bacterial relative abundance values were analyzed using Tukey’ test. a, b, c Means with different superscripts differ significantly each row.*

### Analysis of Bacterial Composition in Omasum

It was observed that in omasum, the prokaryotic communities were dominated by the phyla Bacteroidetes (33.20%), Proteobacteria (15.62%) at the phylum level on Day 3. Interestingly, the Bacteroidetes (54.51%) had increased by Day 7, becoming the most abundant phylum in the samples collected after Day 7 (**Figure 1A** and **Supplementary Table S4**). At days 3 through 56, the populations of Proteobacteria exhibited a gradual but decrease with age. By contrast, the higher annotated reads (21.15% and 1.72%) belonged to Fusobacteria at days 3 and 7, respectively (**Figure 1A**). At the genus level, the majority of the reads (21.15% and 17.75%) were classified as *Fusobacterium* and *Bacteroide* at Day 3, *Prevotella_1* *Rikenellaceae_RC9* and *Bacteroidales_S24-7* were observed to exhibit similar changes in the reticulum and omasum (**Supplementary Table S5**). The proportions of *Christensenellaceae_R-7* at Day 28 were higher than at all other ages (*P* < 0.05) (**Table 2**). The proportions of *Ruminococcaceae_NK4A214_group* at Day 14–28 were higher than that at all other stages (*P* = 0.0123) (**Table 2**). Taken together, these data demonstrate the heterogeneous nature of the omasum microbiota at different developmental stages in terms of genus-level composition.

### Analysis of Bacterial Population Composition in Abomasum

At the phylum level, most of the annotated reads (62.10%) belonged to Firmicutes, followed by Bacteroidetes (24.15%) at Day 3. As the ages of the animals increased, the predominant phylum became Bacteroidetes (**Figure 1A**). Between days 3 and 56, the observed low abundance of the phylum Fusobacteria gradually decreased with age (**Supplementary Figure S3**). In contrast, Spirochaetae gradually increased with age. The proportion of Verrucomicrobia at Day 28 was greater than at all other ages (**Supplementary Figure S3**). At the genus level, *Bacteroides*, *Porphyromonas*, *Prevotella_1*, *Rikenellaceae_RC9* were observed to follow similar patterns of population dynamics in the reticulum (**Supplementary Figure S4**). Extraordinary, the proportion of *Conchiformibius* was observed to account for 3.57% at Day 3, whereas after Day 3, it only accounted for less than 0.34% (**Table 2**). The proportion of *Lactobacillus* (52.87%) at Day 3 was greater than at all other ages. The proportion of *Ruminococcaceae_NK4A214* at Day 14 was higher than at all other ages (*P* < 0.001) (**Table 2**). In ruminants, the abomasum is the last compartment of the multi-chambered stomach and serves as the gastric stomach where acid is secreted, and digestion as it occurs in non-ruminant mammalian species, begins. These data indicate that the primary role of *Lactobacillus* is to reduce the pH of the abomasum, allowing abomasum to function as a sort of barrier for bacterial transmission to the lower gastrointestinal tract.

### OTU Diversity and Similarity Analysis

Community OTU comparisons by principal co-ordinates analysis (PCoA) of each group using the Bray–Curtis similarity metric revealed that the bacterial populations in each sample were best clustered together according to the age of the hosts, suggesting each group hosts its own distinct bacterial community. The average within-group similarity analysis showed a significant difference between the groups, which increased in an age-dependent manner (**Figure 1B**). Our results also revealed a sub-clustering within the Day 3–14 group, Day 21–28 group, and Day 42–56 group, the community structure was similar among the different of stomach compartments at the same age group (**Figure 1B**). The proportion of Fusobacteria in abomasum was lower than that in reticulum and omasum (**Supplementary Figure S5**). The function of Fusobacteria is mainly cellulose decomposition. At the family level in the abomasum, the proportion of Lactobacillaceae (11.35%) Clostridiales_vadinBB60_group (0.96%), Staphylococcaceae (0.01%), and Planococcaceae (0.03%) were significantly higher than in both the reticulum and omasum samples (**Table 3** and **Supplementary Figure S6**). In samples collected from the omasum, the proportion of Christensenellaceae (3.49%), Acidaminococcaceae (2.14%), Coriobacteriaceae (0.09%), and Peptococcaceae (0.55%) were significantly higher than in the samples collected from the reticulum and abomasum (**Table 3** and **Supplementary Figure S6**). At the genus level in the abomasum, the proportions of *Lactobacillus* (11.35%), *Clostridiales_vadinBB60* (0.96%), and *Jeotgalicoccus* (0.01%) were significantly higher than in reticulum and omasum samples (**Table 3**). In the omasum, the proportions of *Peptococcaceae* (0.51%), *Christensenellaceae_R-7_group* (3.45%), *Lachnospiraceae_UCG-008* (0.07%), *Lachnospiraceae_UCG-010* (0.22%), and *Anaerotruncus* (0.05%) were significantly higher than in samples collected from either the reticulum or abomasum (**Table 3**).

**Table 3:** Distribution differences at the family and genus level in goat compound stomachs.

Species	Reticulum (%)	Omasum (%)	Abomasum (%)	*P*-value
f_Christensenellaceae	2.118^ab^	3.490^a^	0.952^b^	0.004
f_Lactobacillaceae	0.760^a^	1.720^a^	11.350^b^	0.040
f_Acidaminococcaceae	1.486^ab^	2.143^a^	0.827^b^	0.010
*g_Clostridiales_vadinBB60*	0.265^b^	0.068^b^	0.962^a^	0.003
*g_Christensenellaceae_R-7*	2.096^ab^	3.456^b^	0.947^a^	0.004
*g_Lactobacillus*	0.760^b^	1.721^b^	11.350^a^	0.040
*g_Erysipelotrichaceae*	0.285^b^	0.228^ab^	0.041^a^	0.002
*g_Jeotgalicoccus*	0.001^b^	0.002^b^	0.010^a^	0.011
*g_Anaerotruncus*	0.054^b^	0.050^b^	0.009^a^	0.001
*g_Succiniclasticum*	1.249^ab^	1.829^b^	0.705^a^	0.038
*g_Streptococcus*	0.371^ab^	0.682^b^	0.096^a^	0.005
*g_Lachnospiraceae_UCG-010*	0.120^ab^	0.226^b^	0.040^a^	0.003

*The bacterial relative abundance values were analyzed using Tukey’ test. a, b, c Means with different superscripts differ significantly each row.*

### Within-Network Interactions Mirrored the Bacterial Microbiota Relationships

**FIGURE 2:**
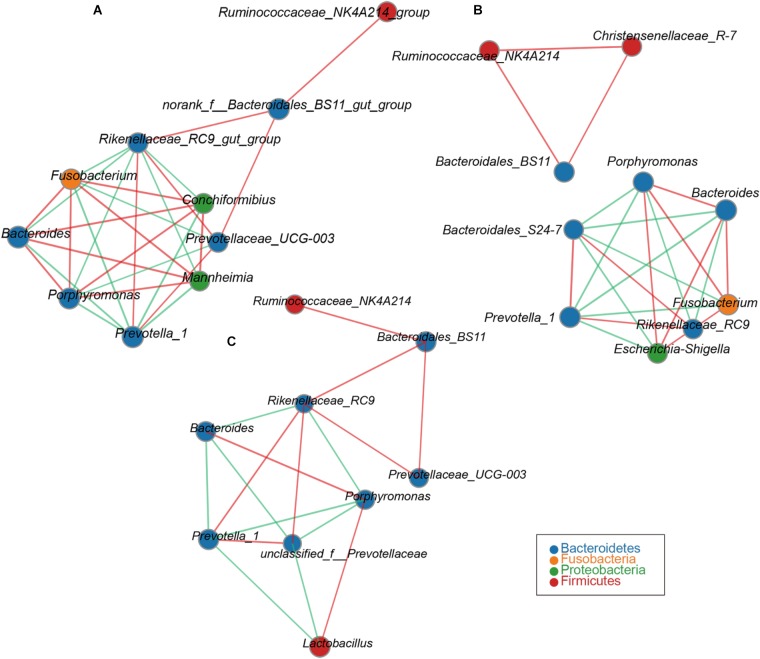
Network analysis applied to the goat compound stomach. Reticulum **(A)**, omasum **(B)**, abomasum **(C)** microbiota at different ages. Genus correlation network maps mainly reflect the genus-relatedness of each taxonomic level under an environmental condition. By calculating the correlation coefficient between genera to reflect the correlation between genera. The size of the node is proportional to the abundance of the genera. Node color corresponds to phylum taxonomic classification. Edge color represents positive (green) and negative (red) correlations, and the edge thickness is equivalent to the correlation values.

Network analysis was used to analyze the correlation between species abundance among different samples in this study. This would provide further insights into the mechanism of the formation of phenotypic differences between the samples. The reticulum network contains a node representing *Prevotella_1*. Its neighbors form seven mutually exclusive clusters: two positively correlated with *Prevotella_1*, consisting mainly of members of the *Prevotellaceae_UGG-03* and *Rikenellaceae_RC9*. The other was negatively correlated with *Prevotella_1*, consisting mainly of *Bacteroides*, *Porphyromonas*, *Fusobacterium*, *Conchiformibius*, *Mannheimia* (**Figure 2A**). The omasum network contains a node representing *Porphyromonas*. Its neighbors form six mutually exclusive clusters: three were positively correlated with *Porphyromonas*, consisting mainly of members of the *Escherichia-Shigella*, *Fusobacterium* and *Bacteroides*, and the other negatively correlated with *Porphyromonas*, consisting mainly of members of the *Prevotella_1*, *Rikenellaceae_RC9*, and *Bacteroidales_S24-7* (**Figure 2B**). The genus of *Bacteroidales_BS11* was observed to be positively correlated with *Ruminococcaceae_NK4A214* and *Christensenellaceae_R-7* (**Figure 2B**). The network observed within the abomasum contains a node representing *Rikenellaceae_RC9*. Its neighbors form 6 mutually exclusive clusters. Four were positively correlated with *Rikenellaceae_RC9*, consisting members of *Bacteroidales_BS11*, *Prevotellaceae_UGG-03*, *Prevotella_1*, and *Prevotellaceae*. The remaining cluster was negatively correlated with *Rikenellaceae_RC9*, consisting mainly of members of the, *Bacteroides* and *Porphyromonas* (**Figure 2C**).

### Predicted Molecular Functions of Bacterial Microbiota

**FIGURE 3:**
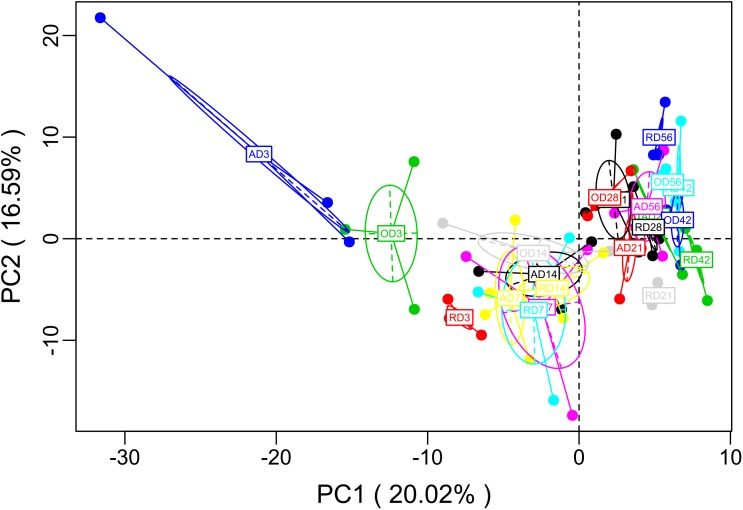
PCA of microbial functional diversity across compound stomach samples. After the dimensionality analysis of the sample, there are relative coordinate points on the principal component, the distance of each sample point represents the distance of the sample, and the sample in the same area in the plane shows the similarity (A: abomasum; O: omasum; R: reticulum).

To better understand the molecular functions of the bacterial microbiota across goat compound stomachs, PICRUSt was used to predict likely functions. A total of 278 gene families were identified in the three groups. Of the 278 gene families, most of the genes belonged to Transport (4.97% in reticulum, 5.31% in omasum, 4.96% in abomasum), DNA_repair_and_recombination_proteins (3.07% in reticulum, 3.04% in omasum, 3.15% in abomasum), Ribosome (2.72% in reticulum, 2.64% in omasum, 2.82% in abomasum), Purine metabolism (2.38% in reticulum, 2.36% in omasum, 2.52% in abomasum), and ABC transporters (2.54% in reticulum, 2.75% in omasum, 2.48% in abomasum). A principal component analysis (PCA) on the relative abundance values of the KEGG pathways represented from the different age group microbiota showed a clear distinction (**Figure 3**). Genes related to Glycolysis/Gluconeogenesis, Pyruvate metabolism, Aminoacyl_tRNA_biosynthesis, DNA repair and recombination proteins, Pyrimidine metabolism, and Purine metabolism were compared among the different age groups, and were observed to be enriched at Day 3 in abomasum (**Supplementary Figure S7**). The abundance of genes related to ABC transporters, Transporters, Transcription factors, and Other ion coupled transporters were enriched at Day 3 in the omasum (**Supplementary Figure S7**). These results suggest that the microbiota at Day 3 is primarily responsible for glucose transport, converting starch and sucrose into glucose and fructose through the enzymatic activities. In addition, the microbiota genes expressed at Day 3 serve to accelerate the conversion between Glycerate-2P and Phosphoenol-pyruvate.

## Discussion

**FIGURE 4:**
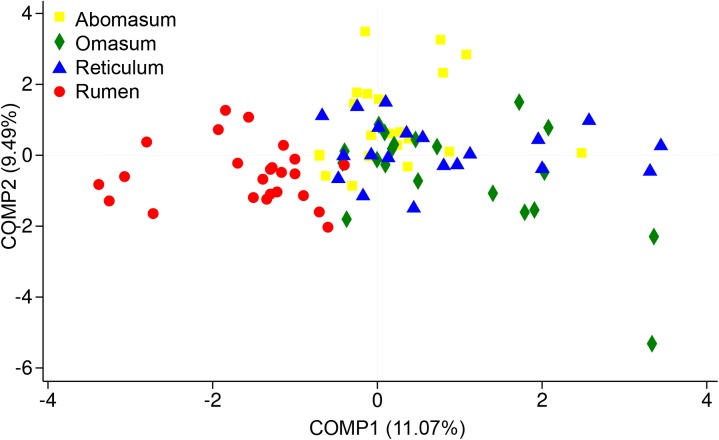
Partial least squares discriminant analysis (PLS-DA) scores plots of the microbiota in four stomachs based on sequencing at the phylum level. PLS-DA is a partial least squares regression of a set of Y of binary variables describing the categories of a categorical variable on a set X of predictor variables. It is a compromise between the usual discriminant analysis and a discriminant analysis on the significant principal components of the predictor variables.

In the present study, we sought to characterize the systematic microbiota dynamics in the compound stomachs of ruminants during the transition from birth to weaning, as well as to determine the compositional changes in the colonizing bacterial populations during early development. Our findings suggest that each sampled age group has its own distinct microbiota, which is reflected by the clustering of the samples by age group (**Figure 1**). In general, the microbiota in compound stomachs undergoes developmental changes that are independent of diet in pre-weaned goats. By combing our recent rumen bacterial population composition data (Zhang et al., 2018, unpublished), it was determined that the rumen microbial community exhibited clear spatial differences compared with the other three compartments (**Figure 4**). In contrast to the rumen, the other three stomach compartments exhibited larger temporal differences in the microbiota communities and smaller spatial differences (**Figure 4**).

Previous studies reported that the reagent and laboratory contamination may critically affect 16S rRNA gene sequencing and metagenomic results (Salter et al., 2014). To guarantee the stomach fluid DNA samples were not contaminated by reagents used during the DNA extraction process, NC samples (mainly DNA-free water and buffer used for DNA extraction) were subjected to PCR amplification and sequencing (**Supplementary Figure S1**). Consistent with previous reports (Kennedy et al., 2014; Lauder et al., 2016; Lim et al., 2018), we confirmed the microbial diversity in the NC samples (**Supplementary Table S3**). However, we demonstrate that the microbial diversity in NC only had an effect to the stomach samples with a low microbe density (**Supplementary Table S3**), indicating the microbes in control samples should be considered in metagenomic studies. Based on the observed differences between the newborn animals (Day 3–7) and their low similarity when compared with other age groups (**Figure 2**), we propose that only a few genera are shared between the microbiota communities during the primary stages of colonization and those found in mature animals. This could in part be due to the fact that a pre-functioning rumen is unavailable to digest plant mass during first days of life (Jami et al., 2013). The diversity and richness of OTU numbers increased with the age. This could be explained by the animal’s environment and colostrum intake being contributing factors to the varied diversity of the gastrointestinal microbiota. There is increasing evidence that milk is essential for the initial development of newborns, as it represents a great source of commensal bacteria (Khodayar-Pardo et al., 2014). It is assumed that mature milk is the main postnatal source of bacteria for the infant’s intestines, and thus serves an important role in microbiota colonization after birth (Martín et al., 2003). The data presented here show that of the microbiota flora in the four stomachs is age specific from the 1st week after birth, with Proteobacteria and Fusobacteria detected as the dominant phyla (**Figure 1**).

Previous studies have shown the microbiota in the neonatal gut is of particular interest, because it reflects not only the fragile structure of the bacterial communities, but also the true origin of mammalian gut microbiota. Bacterial communities in the neonatal gut are unstable due to the nature of the system’s rapid temporal variations. Due to the unique abundance of oxygen in the neonatal gut, the microbiota during the first week of life is frequently dominated by facultative anaerobes, mainly Proteobacteria species (Guaraldi and Salvatori, 2012). Other studies have reported that Proteobacteria in the neonatal gut may be derived from the maternal placenta through fetal swallowing of amniotic fluid *in utero* (Koren et al., 2012; Shin et al., 2015). In contrast, beyond the 28th day of life, the gut microbiota is composed almost exclusively of the genus *Prevotella* (**Table 1**). The most abundant bacteria in goats belong to two phyla, Firmicutes and Bacteroidetes. Of the members in Bacteroidetes, two genera dominate-*Bacteroides* and *Prevotella*. *Prevotella* are more common in ruminants who consume a fiber-rich diet (Kovatcheva-Datchary et al., 2015; Ley, 2016). A previous report demonstrated a positive effect on host metabolism by the *Prevotella*-dominated gut microbiota (Kovatcheva-Datchary et al., 2015). In fact, *Prevotella* is capable of metabolizing dietary fiber from plant cell walls, and thus produces significant amounts of short chain fatty acids (SCFAs) that are later absorbed by the host (Ramayo-Caldas et al., 2016). Previous study found that *Prevotella* and *Clostridium* have significantly different cellulose degrading modes, *Prevotella* would preferentially degrade hemicellulose, and when xylan compounds then become accessible, colonization of the fiber surface is gradually taken over by *Clostridium* (Rubino et al., 2017). Therefore, the rapid increase of *Prevotella* in the gastrointestinal tract has a positive effect for the animal’s physiological development. Similarities in the composition of the microbiota were observed between the segments of the four stomach compartments. With increasing age, the same patterns of flora colonization were observed in the reticulum, omasum, and abomasum. This is in agreement with previous reports in which the microbiota within the four stomachs as the digesta passes from one segment to another (Mao et al., 2015; Wang et al., 2017). Lactobacillaceae revealed a higher proportion (11.35%) in abomasum (**Supplementary Figure S6**), whereas Lactobacillaceae accounted for a higher proportion (9.80%) in rumen at Day 56. It has been well established that kids mainly digest in the abomasum, and the rumen begins to share the digestive function until Day 28. Therefore, Lactobacillales may be more abundant in the main digestive organs of the body. An abundance of Lactobacillales has been implicated in arthritis, rheumatic disease, and diabetes (Yeoh et al., 2013; McLean et al., 2015). Furthermore, the relative abundance of Lactobacillales was predictive of higher host T-helper cell counts, suggesting an important link between Lactobacillales and host adaptive immunity (Snijders et al., 2016).

In summary, our results suggest that the microbial community in compound stomachs of ruminants at early ages shifts toward the mature ruminant state, the rumen microbiota community showed strong specificity compared to the other three stomach compartments. Together with our previously published data on rumen colonization (Zhang et al., 2018, unpublished), we show that the microbiome landscape represents three major mature stages in ruminant stomachs compartments early in life. We termed this period the primary stage (the first two weeks after birth) for the gain of access to foreign microorganisms, the secondary stage (Days 14–28) is the period of microbial transition, and the third stage (after Day 28) is the exogenous and endogenous microbial colonization stage. The diversity and within-group similarity increased with age, suggesting a more diverse but homogeneous and specific mature community, relative to the more heterogeneous and less diverse primary community. The findings presented here provide a clearer picture of the biochemical and microbial functions within the gastrointestinal tract in ruminants. Furthermore, the data provide additional insights into diet formulation to promote ruminant animal health during the early stages of development.

## Author Contributions

KZ, YL, MG, YY, XW, and YC designed and conceived the experiments. KZ, YL, GL, and BL performed the experiments. KZ, YL, CL, and MG carried out microbial data processing, analysis, and interpretation. KZ and XW wrote the manuscript.

## Conflict of Interest Statement

The authors declare that the research was conducted in the absence of any commercial or financial relationships that could be construed as a potential conflict of interest.

## Footnotes

1 https://github.com/benjjneb/decontam

2 http://drive5.com/uparse/

3 www.i-sanger.com

4 http://rdp.cme.msu.edu/

5 http://fungene.cme.msu.edu/

## Supplementary Material

FIGURE S1 Quality control of the microbial DNA extracted from stomach fluid samples. **(A)** Microbial DNA extracted with the EZNA^®^ Stool DNA kit, NC: the mixture of reagents and water used. **(B)** Microbial DNA were extracted with the Fast-DNA^®^ Spin kit for soil. NC: the mixture of reagents and water used. Amplified PCR products using the DNA extracted from rumen **(C)**, and abomasum and reticulum **(D)** fluid samples.

FIGURE S2 Summary of rarefaction results based on operational taxonomic units (OTUs) (3% divergence) for each sample.

FIGURE S3 Phylum level analysis of goat abomasum bacteria in different age groups. The ordinate indicates the species name under different classification levels, and the abscissa indicates the percentage of the abundance of a species of the sample. Different colors represent different groups.

FIGURE S4 Genus level analysis of goat abomasum bacteria in different age groups. The ordinate indicates the species name under different classification levels, and the abscissa indicates the percentage of the abundance of a species of the sample. Different colors represent different groups (^∗^0.01 < *p* ≤ 0.05, ^∗∗^0.001 < *p* < 0.01, ^∗∗∗^*p* ≤ 0.001).

FIGURE S5 Phylum level analysis of goat stomach compartment bacteria. The ordinate indicates the species name under different classification levels, and the abscissa indicates the percentage of the abundance of a species of the sample. Different colors represent different groups.

FIGURE S6 Family level analysis of goat stomach compartment bacteria. The ordinate indicates the species name under different classification levels, and the abscissa indicates the percentage of the abundance of a species of the sample. Different colors represent different groups (^∗^0.01 < *p* ≤ 0.05, ^∗∗^0.001 < *p* < 0.01, ^∗∗∗^*p* ≤ 0.001).

FIGURE S7 Metagenomic functional predictions for samples. Variations in KEGG metabolic pathways in functional bacterial communities throughout goat rumens.

TABLE S1 Ingredients and nutrients of the experimental diets.

TABLE S2 The sequence information of 63 different group sample.

TABLE S3 The OTU classification statistics of the negative control sample.

TABLE S4 The dynamics of bacterial community at the phyla level in different treatment groups.

TABLE S5 The changes of bacterial community at the genus level in different treatment groups.
