# Supplementary figures and images for: Exploring the Spatial-Temporal Microbiota of Compound Stomachs in a Pre-weaned Goat Model
Source: Front Microbiol. 2018 Aug 15;9:1846. doi: 10.3389/fmicb.2018.01846 (PMC6104157; doi:10.3389/fmicb.2018.01846)

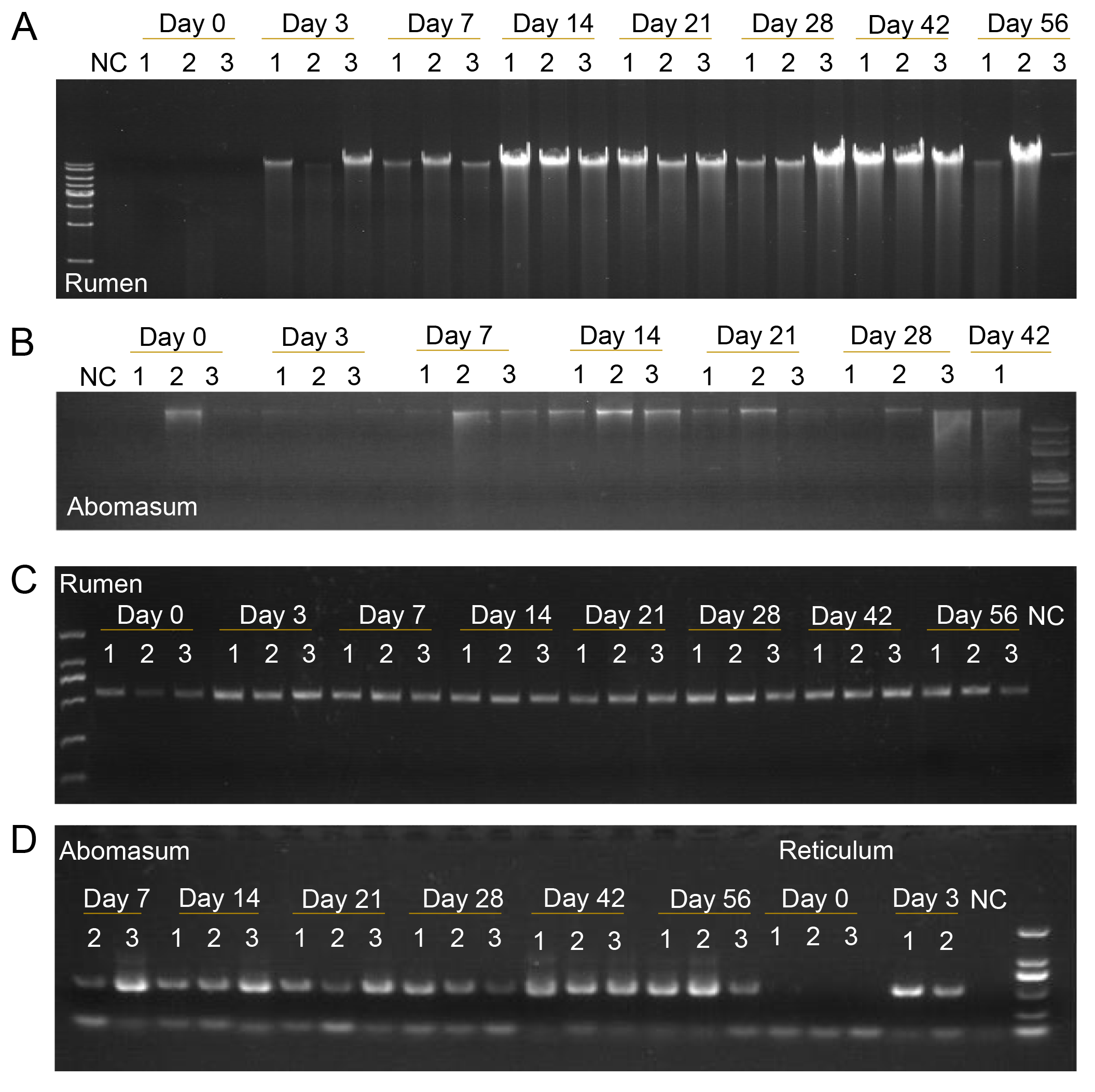

Supplement: FIGURE S1 — Quality control of the microbial DNA extracted from stomach fluid samples. (A) Microbial DNA extracted with the EZNA® Stool DNA kit, NC: the mixture of reagents and water used. (B) Microbial DNA were extracted with the Fast-DNA® Spin kit for soil. NC: the mixture of reagents and water used. Amplified PCR products using the DNA extracted from rumen (C), and abomasum and reticulum (D) fluid samples. [file Image_1.TIF]

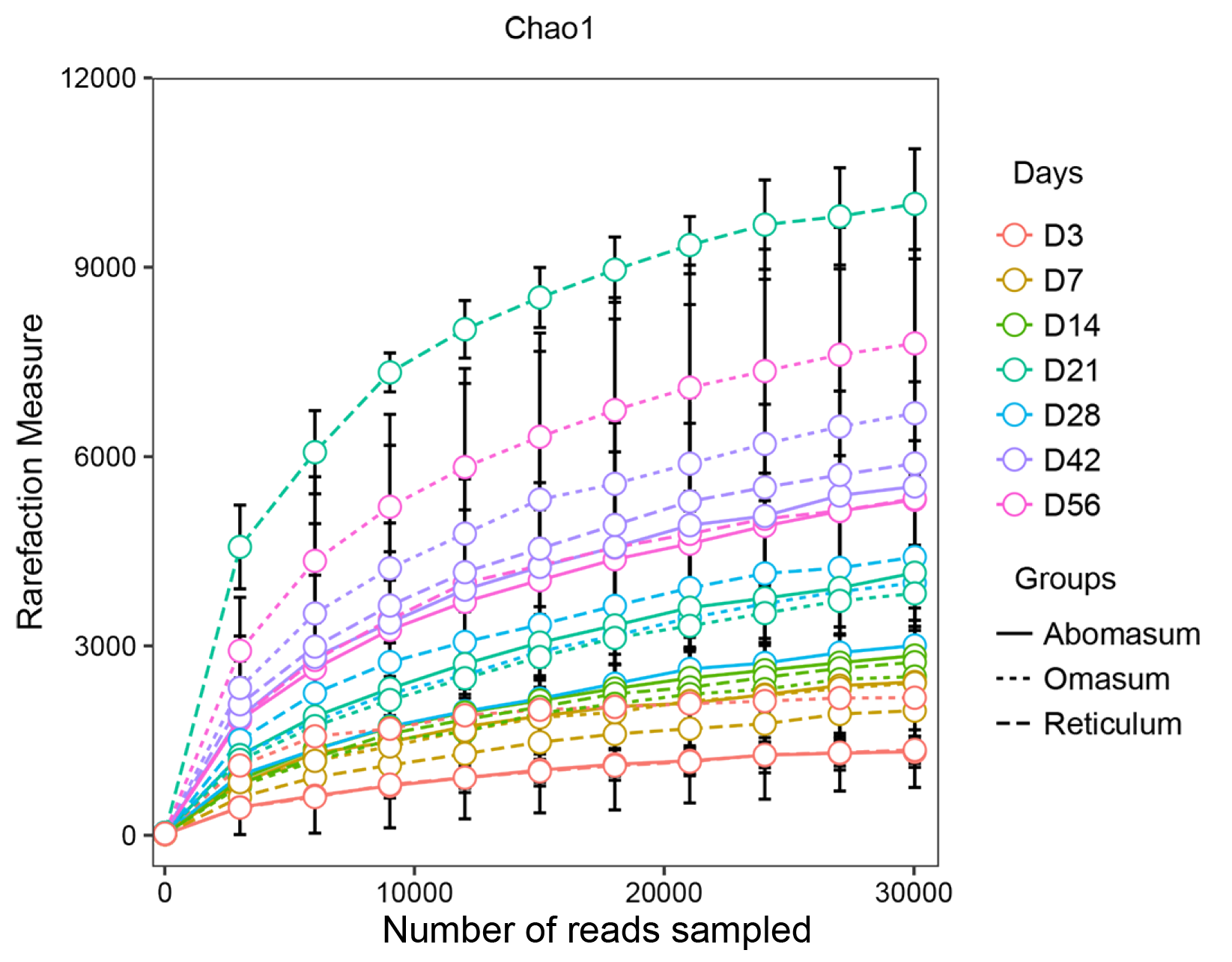

Supplement: FIGURE S2 — Summary of rarefaction results based on operational taxonomic units (OTUs) (3% divergence) for each sample. [file Image_2.TIF]

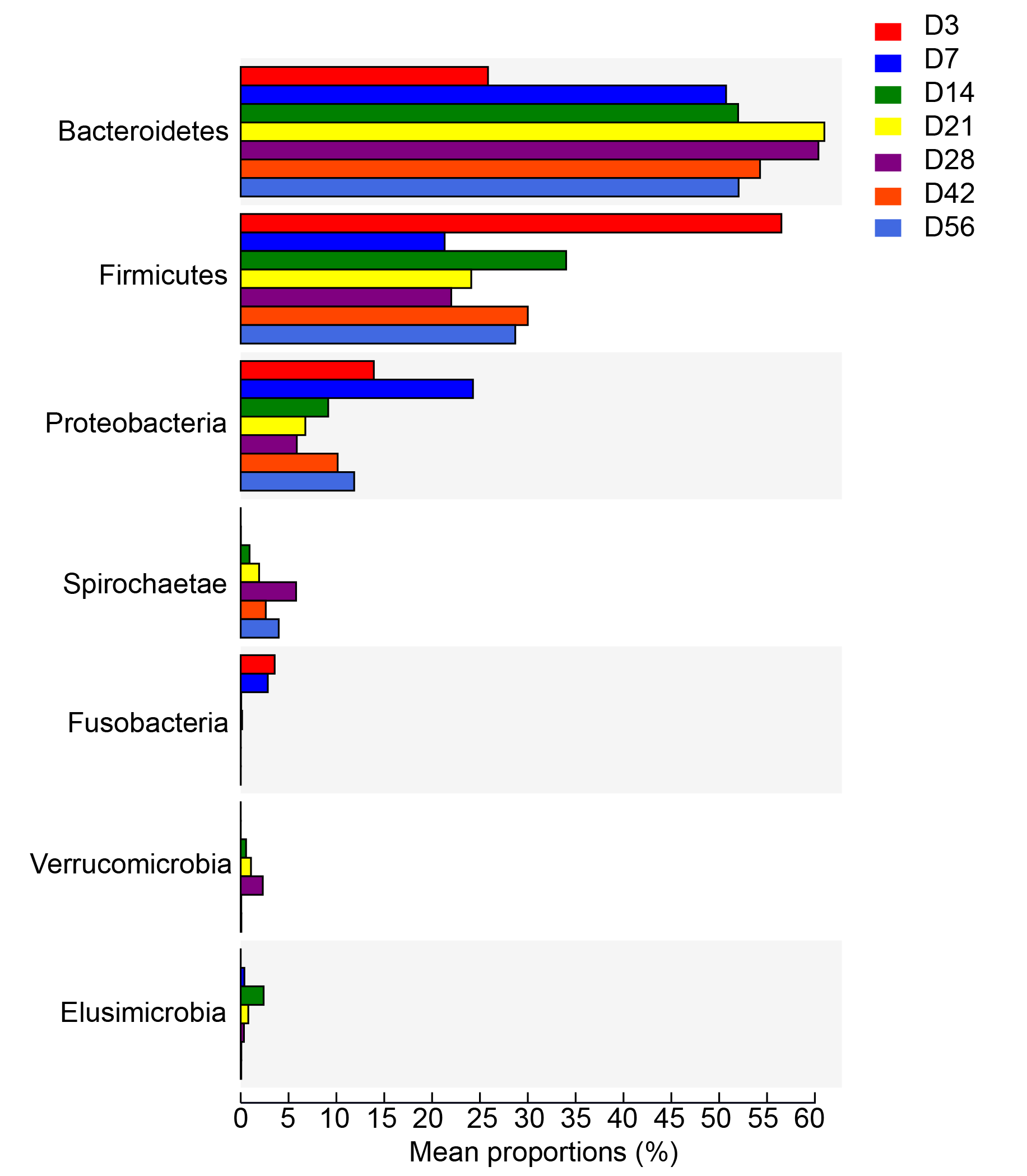

Supplement: FIGURE S3 — Phylum level analysis of goat abomasum bacteria in different age groups. The ordinate indicates the species name under different classification levels, and the abscissa indicates the percentage of the abundance of a species of the sample. Different colors represent different groups. [file Image_3.TIF]

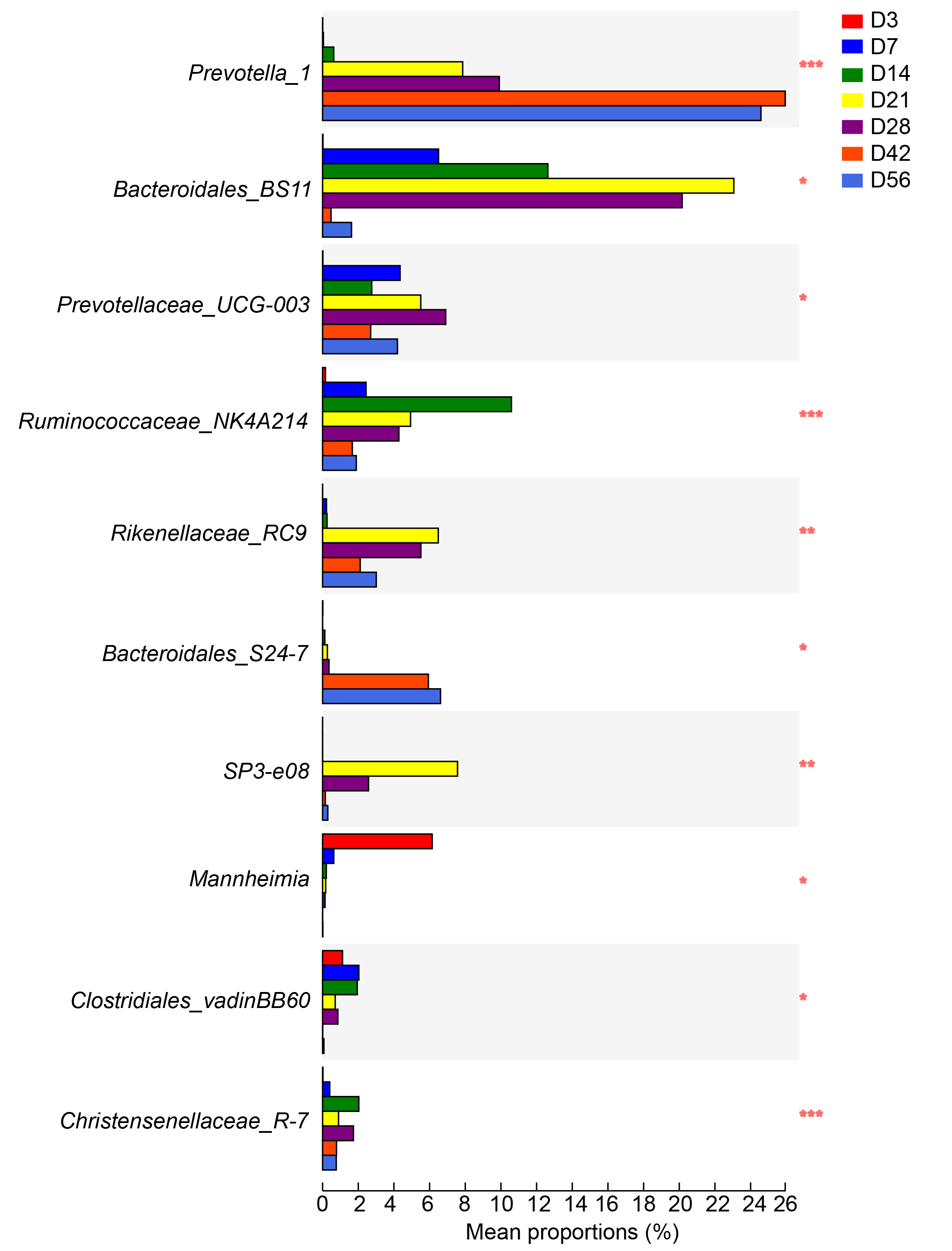

Supplement: FIGURE S4 — Genus level analysis of goat abomasum bacteria in different age groups. The ordinate indicates the species name under different classification levels, and the abscissa indicates the percentage of the abundance of a species of the sample. Different colors represent different groups (∗0.01 < p ≤ 0.05, ∗∗0.001 < p < 0.01, ∗∗∗p ≤ 0.001). [file Image_4.TIF]

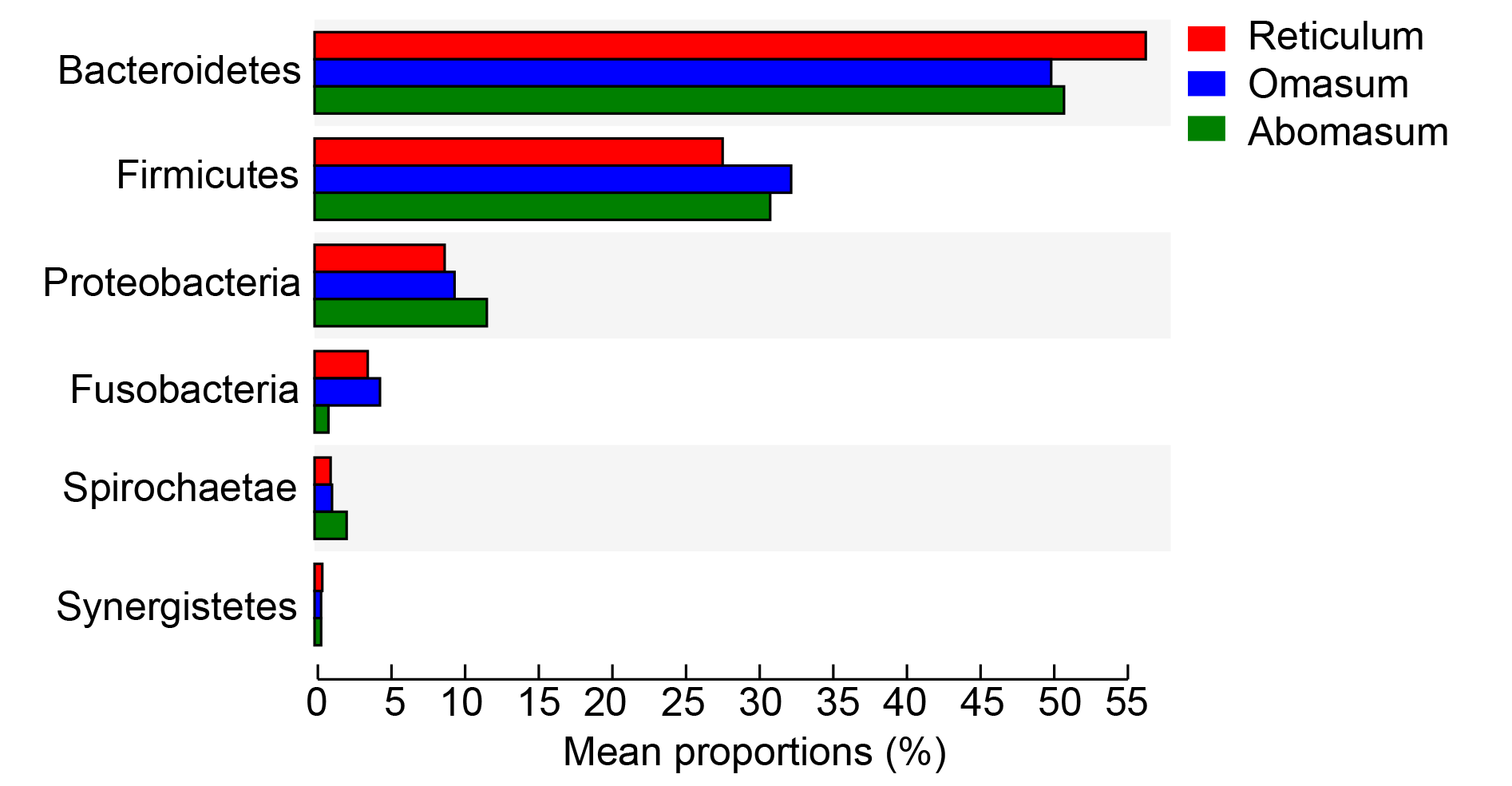

Supplement: FIGURE S5 — Phylum level analysis of goat stomach compartment bacteria. The ordinate indicates the species name under different classification levels, and the abscissa indicates the percentage of the abundance of a species of the sample. Different colors represent different groups. [file Image_5.TIF]

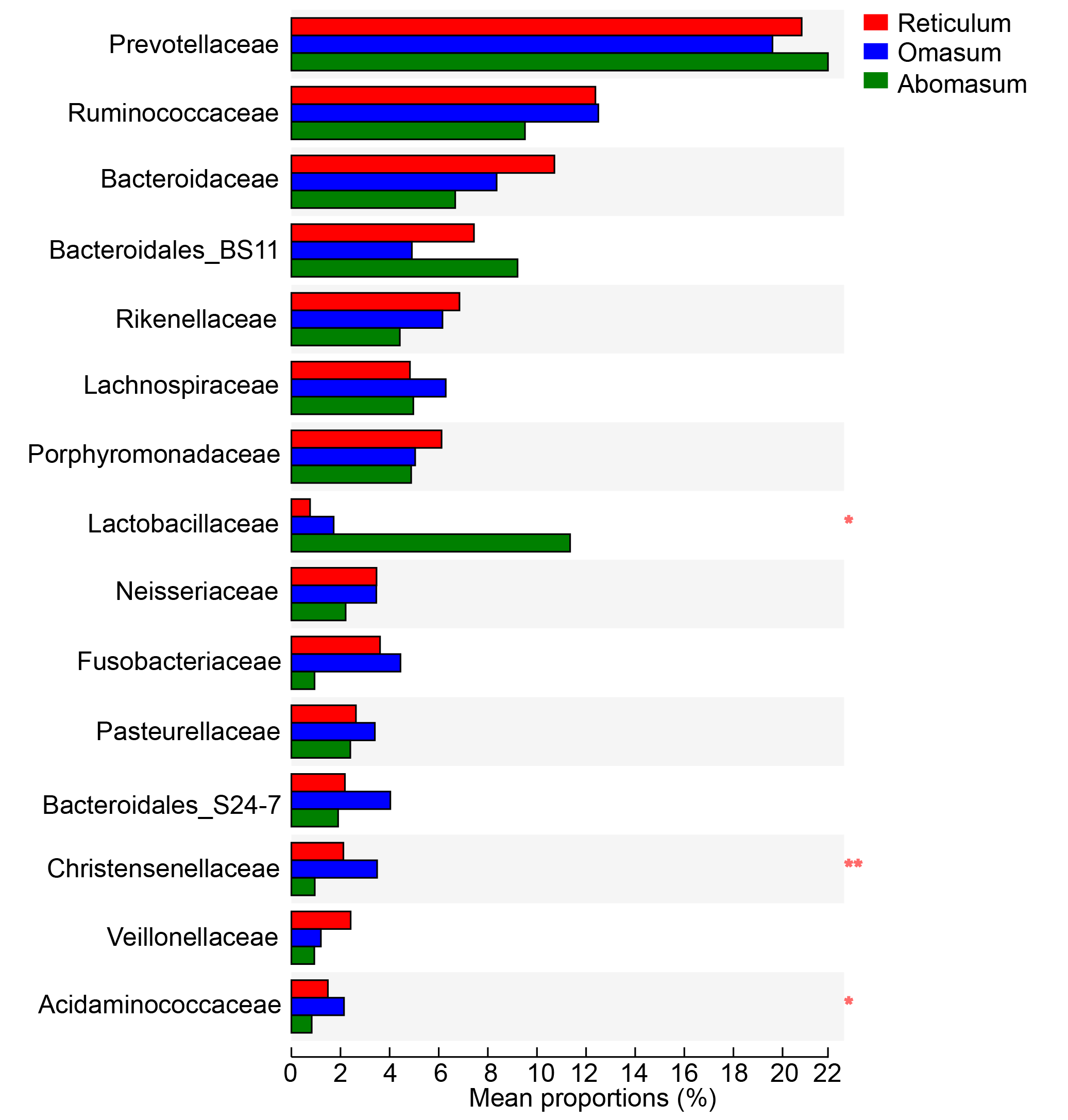

Supplement: FIGURE S6 — Family level analysis of goat stomach compartment bacteria. The ordinate indicates the species name under different classification levels, and the abscissa indicates the percentage of the abundance of a species of the sample. Different colors represent different groups (∗0.01 < p ≤ 0.05, ∗∗0.001 < p < 0.01, ∗∗∗p ≤ 0.001). [file Image_6.TIF]

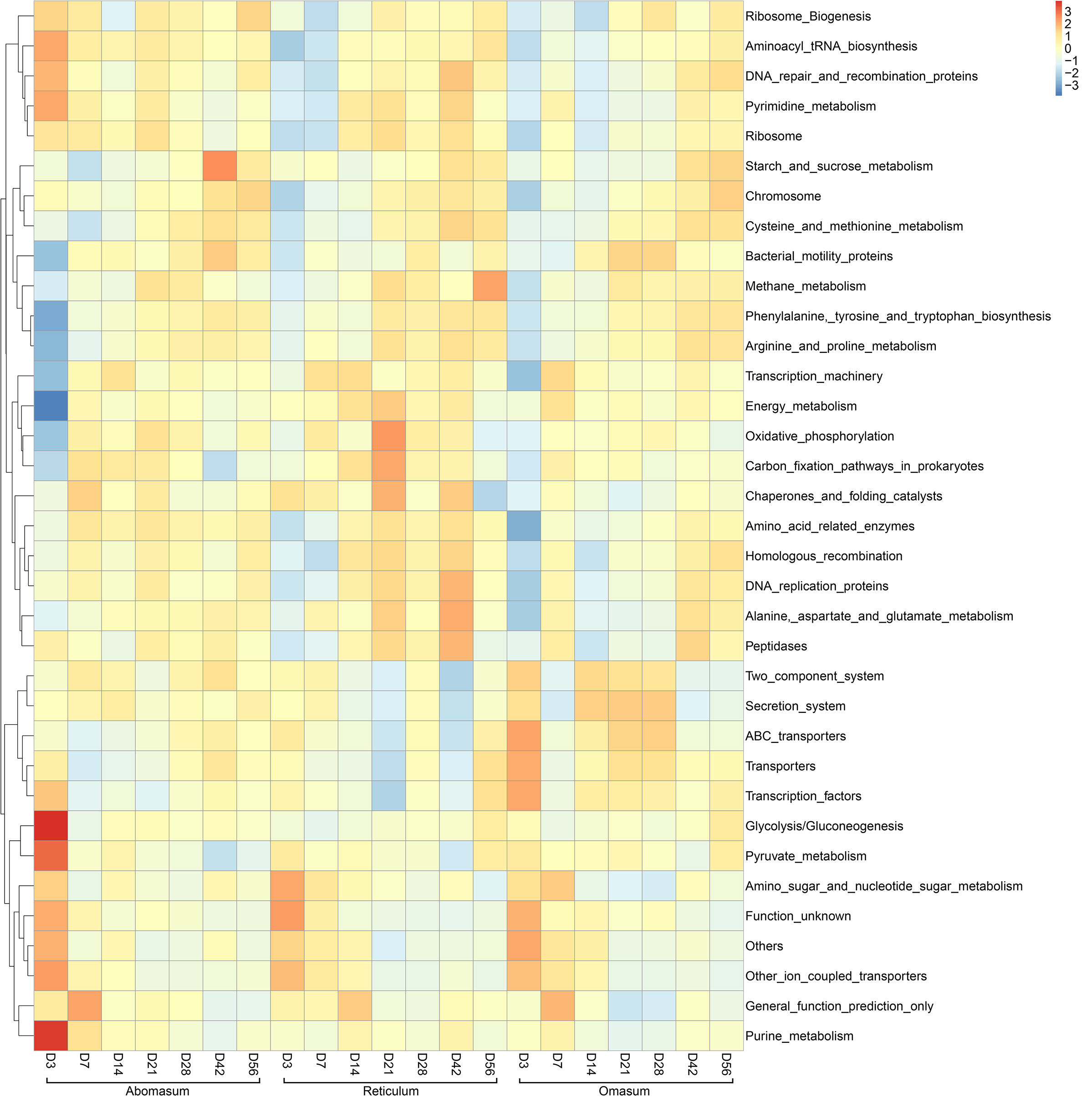

Supplement: FIGURE S7 — Metagenomic functional predictions for samples. Variations in KEGG metabolic pathways in functional bacterial communities throughout goat rumens. [file Image_7.TIF]
